# Acute Kidney Injury After Peripheral Interventions Using Carbon Dioxide Angiography—Risk Factors Beyond Iodinated Contrast Media

**DOI:** 10.3390/life15071046

**Published:** 2025-06-30

**Authors:** Tim Wittig, Sarah Fischer, Birte Winther, Andrej Schmidt, Dierk Scheinert, Anne Hoffmann, Sabine Steiner

**Affiliations:** 1 Department of Angiology, University Hospital Leipzig, 04103 Leipzig, Germany; birte.winther@medizin.uni-leipzig.de (B.W.); andrej.schmidt@medizin.uni-leipzig.de (A.S.); dierk.scheinert@medizin.uni-leipzig.de (D.S.); 2 Helmholtz Institute for Metabolic, Obesity and Vascular Research (HI-MAG), Helmholtz Center Munich, University of Leipzig, University Hospital Leipzig, 04103 Leipzig, Germany; anne.hoffmann@helmholtz-munich.de (A.H.); sabine.m.steiner@meduniwien.ac.at (S.S.); 3 Clinic for Geriatrics, Sachsenklinik Naunhof, 04683 Naunhof, Germany; 4 Division of Angiology, Department of Medicine II, Medical University Vienna, 1090 Vienna, Austria

**Keywords:** chronic kidney disease, peripheral artery disease, post-contrast acute kidney injury, carbon dioxide angiography, iodinated contrast medium

## Abstract

Contrast-associated acute kidney injury (CA-AKI) is a known complication of endovascular procedures using an iodinated contrast medium (ICM), especially in patients with peripheral artery disease (PAD) and chronic kidney disease (CKD). This retrospective study evaluated the incidence and risk factors of AKI in patients with PAD and CKD undergoing diagnostic angiography or endovascular intervention using carbon dioxide (CO_2_) as the primary contrast medium, with optional bailout ICM use. We included 340 patients who underwent peripheral angiography or intervention between September 2014 and December 2020. CO_2_ was used as the primary contrast medium for all patients, as the majority were classified with advanced CKD stages 3–5 according to the Kidney Disease: Improving Global Outcomes (KDIGO) guidelines. Bailout ICM was used in 80% of cases (mean 21.23 ± 14.09 mL). Postinterventional AKI occurred in 13.2% of patients, with over 70% classified as stage 1. Seven patients required new dialysis within 7 days. Multivariate analysis identified hypertension, heart failure, and coronary artery disease as independent AKI risk factors. Statin or Renin–Angiotensin–Aldosteron System (RAAS) inhibitor use and higher pre-interventional GFR were protective. AKI remains common in patients undergoing CO_2_-guided peripheral procedures. Further studies are needed to explore underlying mechanisms and outcomes.

## 1. Introduction

Endovascular revascularization for symptomatic peripheral arterial disease has increased in recent decades due to its minimally invasive nature, enabling faster recovery and fewer perioperative complications [1]. An aging population and the rising prevalence of diabetes have led to an increase in patients presenting with critical limb ischemia, the most severe form of peripheral artery disease. Treating critical limb ischemia often requires complex, multi-vessel infrainguinal revascularization. Endovascular interventions are becoming a preferred alternative to open bypass surgery for managing these challenging situations, but they frequently require significant volumes of iodinated contrast media (ICM).

However, ICM administration is a known risk factor for acute kidney injury (AKI) and major adverse kidney events, including persistent renal impairment, new-onset dialysis, and death [2]. Studies have linked contrast-associated AKI (CA-AKI) with higher in-hospital and long-term adverse events, as well as longer hospital stays and increased costs, primarily in coronary interventions [3,4,5,6,7,8]. In peripheral artery disease patients undergoing peripheral vascular interventions, the incidence of post-procedural AKI is around 10%, though it varies due to factors like patient characteristics, pre-existing chronic kidney disease (CKD), and differing AKI definitions [9]. The incidence and risk factors for CA-AKI following peripheral vascular interventions remain debated, with limited research specifically addressing CA-AKI in peripheral artery disease patients. This gap is significant, as peripheral artery disease patients already have a poor prognosis due to high cardiovascular morbidity and mortality, which may be further exacerbated by CA-AKI [10]. Only recently, the first dedicated scoring system to predict the risk of CA-AKI in patients with peripheral artery disease undergoing peripheral vascular interventions was developed and validated based on pre-procedural characteristics [4], while such scoring systems for patients undergoing coronary interventions have been available for several years [11].

Carbon dioxide (CO_2_) is an alternative to ICM during peripheral vascular interventions, especially in patients with advanced CKD, ICM hypersensitivity, or hyperthyroidism. However, even with minimal or no ICM, a significant number of patients still develop AKI after peripheral vascular interventions [12], and knowledge of its risk factors and consequences remains limited [13]. This study aims to describe AKI occurrence in patients undergoing CO_2_ angiography with no or minimal ICM use and identify associated risk factors and in-hospital adverse events.

## 2. Materials and Methods

Within this retrospective, single-center cohort study, patients with advanced chronic kidney disease (CKD stage 3–5 according to the Kidney Disease: Improving Global Outcomes [KDIGO] guidelines), prior AKI after ICM application, and known ICM allergy or hyperthyroidism were included between 1 September 2014 and 31 December 2020, who underwent diagnostic angiography or endovascular interventions for peripheral artery disease at the Department of Angiology, Leipzig University Hospital (Germany) using CO_2_ angiography. Exclusion criteria comprised the need for pre-interventional dialysis and missing measurements of post-interventional creatinine levels before discharge (Supplemental Figure S1). Comorbidities, cardiovascular risk factors, and medication use were extracted from electronic medical records following standardized collection upon patient admission. Patients provided written informed consent for data collection in a prospectively maintained peripheral artery disease database, and lesion and procedural data were derived from the electronic health records. The Institutional Review Board of the University of Leipzig approved the analysis of this dataset (EK Vote 388/23-ek—23 November 2023), and the study conformed to the principles outlined in the Declaration of Helsinki.

Detailed information on lesion and procedural characteristics was obtained from the intervention report and review of angiographies. Indications for peripheral vascular interventions were based on the recommendations of the European and German guidelines for the treatment of peripheral artery disease [7,14]. Heart rate, oxygen saturation, electrocardiogram, and blood pressure were continuously monitored during the entire procedure. All treatment decisions were at the operators’ discretion, and CO_2_ was used as the main contrast medium (CM) in all interventions. CO_2_ was injected manually in most cases (88.2%) using the CE-certified CO_2_-Angioset system (Optimed Medical Instruments GmbH, Ettlingen, Germany). The Angiodroid Injector (Angiodroid, Italy), the first fully automatic digital injector for peripheral angiography with CO_2_, has been approved by European regulatory authorities and was used for automatic injection. Typically, 20 mL of CO_2_ was injected for iliac and 10 mL for femoropopliteal or infrapopliteal imaging, with adjustments made as clinically needed. In the case of poor image quality or CO_2_ intolerance, additional ICM was administered. Periprocedural complications were noted. After successful peripheral vascular intervention, all patients received platelet inhibition or anticoagulation according to the recommendation in the guidelines at that time from the European Society for Cardiology (ESC) on the diagnosis and treatment of peripheral artery disease, in collaboration with the European Society for Vascular Surgery (ESVS) [14].

Laboratory parameters and the estimated glomerular filtration rate (eGFR) were assessed on admission and within 72 h post-procedure. In the case of AKI, additional laboratory tests were performed as requested by the treating physicians. Creatinine was measured by spectrometry, and eGFR was calculated using the Chronic Kidney Disease Epidemiology Collaboration formula (CKD-EPI). Patients were classified into three stages of CKD based on the KDIGO classification: stage G3: eGFR 30–59 mL/min/1.73 m^2^; stage G4: eGFR 15–29 mL/min/1.73 m^2^; and stage G5: eGFR < 15 mL/min/1.73 m^2^. Postinterventional AKI was defined as a 1.5–1.9× or ≥0.3 mg/dL creatinine increase within 48 h of the procedure for Kidney Injury Network (AKIN) classification stadium I, 2.0–2.9× creatinine increase for AKIN stadium II, and >3.0× or >4 mg/dL creatinine increase or dialysis requirement for AKIN stadium III [15].

Key study endpoints included the incidence of AKI within 7 days post-procedure and adverse events, including death, major bleeding, major amputation, myocardial infarction, and cerebrovascular events until discharge. Descriptive data are presented as numbers (percentages) for categorical data and means (±standard deviation) for continuous data. For categorical variables, Fisher’s exact test was used to compare groups, and Student’s *t*-test or one-way analysis of variance was used for continuous data.

Logistic regression was used to evaluate factors associated with the development of CA-AKI. Due to significant class imbalance (approximately 1:6.6 AKI to non-AKI cases), the random over-sampling examples (ROSE) technique was applied to generate synthetic AKI cases using the ROSE v0.0-4 R package (Version 2024.12.0+467, Posit Software, PBC) (Supplemental Figure S2) [16]. The dataset was divided into training and test sets (a 70% to 30% ratio), ensuring the distribution of the target variable. A repeated cross-validation (five repetitions) utilizing bagged classification techniques was used. A tree bag ensemble model with 50 trees was trained, and performance was evaluated using the Receiver Operating Characteristic (ROC) metric (Supplemental Figure S3). The caret v6.0-94 R package (Version 2024.12.0+467, Posit Software, PBC) was used for model fitting and evaluation [17].

A logistic regression model, a generalized linear model (GLM) with a binomial response and logit link function, was used to assess predictor–target relationships. Multicollinearity was checked using Variance Inflation Factor (VIF) scores, and features were selected for clinical relevance. Analyses were performed using SPSS 29.0 (IBM, Armonk, NY, USA) or R 4.4.1 [16], with *p*-values < 0.05 considered significant.

## 3. Results

### 3.1. Patient and Procedural Characteristics

Over the study period, 340 patients with advanced CKD were identified undergoing diagnostic angiography or endovascular peripheral intervention using CO_2_ angiography with no or only minimal bailout use of ICM. Detailed patient characteristics are given in Table 1, and lesion and procedural characteristics are presented in Table 2. The mean age of patients was 74.3 ± 10.3 years, with approximately 70% male. Most patients exhibited a high cardiovascular risk profile, including high rates of hyperlipidemia (63.5%), hypertension (92.4%), and diabetes (62.1%). Over half suffered from critical limb ischemia, and more than three-quarters reported previous peripheral vascular interventions. Additionally, 94.1% had chronic renal insufficiency stage 3 or worse, with an average GFR of 32.07 ± 15.61 mL/min/1.73 m^2^. In the cohort that developed postinterventional AKI, patients were younger (*p* = 0.019), significantly fewer patients received angiotensin-converting enzyme (ACE) inhibitor or angiotensin receptor blocker (ARB) therapy (*p* = 0.004), and baseline renal function was significantly lower. Additionally, a significantly higher proportion of patients with subsequent AKI presented with CLI (*p* = 0.038).

Most patients (76.2%) underwent femoropopliteal interventions, with CO_2_ angiography primarily indicated by advanced CKD, ICM allergy, prior AKI after ICM application, and hyperthyroidism. Periinterventional hydration was administered in two-thirds of cases. Additional ICM was used in ~80% of cases, mainly due to poor image quality of infrapopliteal segments, with an average volume of 21.23 ± 14.09 mL. Procedural success, defined as residual stenosis <50%, was achieved in 92.3% of cases. Patients who received periprocedural intravenous hydration had significantly lower rates of postinterventional AKI (*p* < 0.001). Additionally, the area–dose product (*p* < 0.001) and the rate of bare metal stent (BMS) implantation (*p* = 0.006) were significantly higher in patients who developed AKI.

### 3.2. Postinterventional AKI, Periprocedural Complications, and Predictors of AKI

Detailed patient characteristics related to postinterventional AKI are presented in Table 3. AKI occurred in 13.2% of cases, with over 70% classified as stage 1 acute renal failure (as per the AKIN classification). The average onset of acute renal failure manifested 2.6 ± 1.76 days post-procedure. De novo dialysis was required in 7 patients within the first 7 days post-exposure. There was no significant difference in patients receiving bailout ICM and no ICM regarding the occurrence of postinterventional AKI (Table 3). Periprocedural complications, major amputations, and 30-day mortality are shown in Table 4. Patients with AKI experienced higher rates of periinterventional myocardial infarction and major amputation within 30 days. Twelve patients died within 30 days, with no deaths considered to be associated with the endovascular procedure. The average length of hospital stay was 7.84 ± 10.94 days. Patients without AKI had a shorter hospital stay (No AKI: 6.46 ± 8.90 vs. AKI: 16.91 ± 17.24; *p* < 0.001). In patients suffering from AKI, the rates of postinterventional myocardial infarctions (*p* = 0.008) and major amputations (*p* = 0.033) were significantly higher.

The logistic regression analysis performed to evaluate factors associated with the development of AKI (Supplemental Figure S1) showed high sensitivity (recall 94.92%) in identifying patients at risk for AKI, but low specificity (23.82%), leading to many false positives. The moderate F1-score (0.636) reflects the balance between precision and recall. The area under the receiver operating characteristic (ROC) curve (AUC: 0.766) indicates acceptable discriminatory ability in distinguishing between patients with and without AKI risk (Supplemental Figure S2).

Significant risk factors for developing AKI included hypertension, congestive heart failure, and coronary artery disease. Protective factors encompassed the use of ACE inhibitors or ARBs, statins, and a higher GFR measured pre-procedure. The volume of additional ICM administered did not have a significant impact, nor did the other tested factors include sex, obesity, smoking, diabetes, nonsteroidal anti-inflammatory drug (NSAID) intake, and critical limb-ischemia status. An overview of these risk and protective factors with odds ratios and confidence intervals is summarized in the Graphical Abstract.

## 4. Discussion

CO_2_ angiography has proven to be a safe and viable alternative to ICM for both diagnostic and therapeutic endovascular procedures, with meta-analyses confirming its nephroprotective effects [18,19]. When performed by experienced interventionalists, it effectively reduces ICM usage. In our large cohort, CO_2_ angiography with minimal ICM bailout maintained sufficient image quality across diverse patient and lesion characteristics. Approximately 20% of the procedures were completed entirely without the use of ICM.

Previous studies have reported higher rates of non-renal complications with CO_2_ angiography, such as nausea, vomiting, and severe abdominal/leg pain [18]. In our cohort, only six (1.8%) patients experienced CO_2_-related complications. This lower incidence may reflect the expertise of the operators, emphasizing the importance of proficiency in the technique. The use of the Angiodroid Injector (Angiodroid, Bologna, Italy) in about 10% of cases likely improved outcomes. This automatic and digital injector offers advantages over manual injection, including better patient tolerability, fewer side effects, and better image quality comparable to ICM-based angiographies [13,20]. Despite standard image post-processing, bailout ICM was used in 80% of cases, mainly due to suboptimal image quality in infrapopliteal segments. Similar findings have been reported in another study, with only 15.7% of patients being treated exclusively with CO_2_ [12]. Bailout ICM was most needed in crural segments with poor outflow, but greater experience with CO_2_ angiography has been shown to reduce additional ICM usage [12].

Despite using CO_2_ and minimal ICM, AKI occurred in 13.2% of cases, usually within 2.6 ± 1.76 days, with over 70% classified as stage 1 acute renal failure, and only 7 patients (2.1%) required dialysis within 7 days. The absence of a significant difference in the incidence of AKI between the group treated with bailout ICM and the group treated with CO_2_ alone suggests that additional factors may contribute to the development of postinterventional AKI. It is important to note that this large cohort included patients with advanced CKD, prior AKI after ICM application, ICM allergy, and hyperthyroidism.

Our findings are consistent with recent studies on AKI incidence after CO_2_ angiography, further highlighting the multifactorial nature of post-intervention AKI beyond ICM use. Jakobi et al. reported an 11.9% AKI rate in a cohort with CKD stage ≥ 3 (KDIGO) and a high critical limb ischemia rate (60%) treated with CO_2_ and bailout ICM [12]. Meta-analyses of heterogeneous cohorts reported AKI incidences of 6.2% [18] and 8.8% [19] after CO_2_ angiography. Regression analysis identified both risk and protective factors for post-procedure AKI in our cohort. These findings partially align with studies on ICM and CO_2_ angiography, suggesting that the mechanisms underlying CA-AKI are not solely dependent on the type of contrast agent used [4,9,12,21,22].

ACE inhibitors/ARBs, statins, and higher pre-interventional GFR were protective against post-intervention AKI. Notably, Renin–Angiotensin–Aldosteron System (RAAS) blockers reduced AKI risk by 62% (OR 0.38), despite prior links to higher CA-AKI risk with ICM exposure. No studies have specifically investigated the role of RAAS blockers in CA-AKI associated with CO_2_ angiography. However, some evidence suggests that ACE inhibitors may reduce ICM-induced AKI by counteracting afferent arteriolar vasoconstriction [23].

Statins consistently reduced AKI incidence following ICM exposure, as also shown in a previous meta-analysis [24]. Statins promote the activity of TGF-β inhibitors, which helps reduce renal fibrosis [24]. Additionally, statins have pleiotropic effects, such as increasing nitric oxide production and providing anti-inflammatory and antioxidative benefits. These effects work together to reduce oxidative stress, cell death (apoptosis), and kidney cell damage after ICM exposure. Some studies also suggest that statin pretreatment before ICM exposure may offer protective benefits [25,26]. Our findings suggest that these protective effects may extend to preventing CA-AKI after CO_2_ exposure, further supporting the role of statins in reducing renal injury risk in high-risk patients.

In our study, hypertension, heart failure, and coronary artery disease were significant risk factors for CA-AKI, consistent with studies in the field [4,9,21,22]. Prasad et al. identified heart failure and diabetes as risk factors for CA-AKI after endovascular therapy in critical limb ischemia patients [9]. Safley et al. found that hypertension, diabetes, and heart failure were independently associated with CA-AKI after peripheral vascular intervention with ICM [4]. Jakobi et al. identified ICM volume as a risk factor for AKI after CO_2_ angiography, with the lowest risk at ≤50 mL [12]. However, in our cohort, additional ICM did not significantly affect AKI incidence. With an average of 21.23 ± 14.09 mL ICM used, the nephrotoxic effects were minimal, supporting CO_2_ angiography with minimal bailout ICM to be safe.

The occurrence of AKI following peripheral vascular intervention involves a complex interplay of protective and risk factors beyond ICM avoidance. Our data underline that AKI is multifactorial, with factors beyond ICM exposure playing a significant role. This supports the need to consider other risk factors in the debate over ICM’s contribution to kidney dysfunction, as some studies question the significant role of ICM altogether [27,28,29] and cite alternative causes like fluid restriction, hypotension, nephrotoxic medications, and hemorrhage [30]. Recently developed predictive models help assess procedural risk based on individual factors in patients with ICM exposure [4]. These models could guide preventive strategies and establish individualized contrast dose limits.

Patients with CA-AKI experienced more cardiovascular events, with higher myocardial infarction and major amputation rates. Previous studies have identified cardiac inflammation, cardiac fibrosis, neurohormonal activation, and electrolyte imbalances following AKI as key contributors to the development of cardiovascular damage [31]. Although usually mild, CA-AKI is associated with worse in-hospital and long-term outcomes, longer stays, and higher costs, suggesting it reflects a high-risk status for cardiovascular events [6,7,8].

Our study has several limitations: it is a single-center, non-randomized trial without a control group using only ICM and lacks long-term follow-up to assess post-intervention AKI’s association with adverse events. Additionally, the statistical model for risk prediction had high sensitivity but low specificity, which may have led to false positives. Further refinement and validation in larger populations are needed.

Future research should focus on enhancing renal protection, refining CO_2_ angiography techniques, and better identifying patients at high risk for post-procedural renal injury. The PERIPREVENT randomized controlled trial (ClinicalTrials.gov ID: NCT06656988) will investigate the role of ICM and CO_2_ in CA-AKI in patients with mildly to severely reduced renal function (KDIGO G3a-b). This study, evaluating a contrast-saving strategy using an automated CO_2_ injection system, will be pivotal in advancing our understanding of the relationship between ICM exposure and AKI.

## 5. Conclusions

In this large study cohort, AKI remained a common sequela following CO_2_ angiography. While the volume of bailout ICM did not appear to predict post-intervention AKI, other risk and protective factors were identified, emphasizing the complex interplay of these variables.

## Figures and Tables

**Table 1:** Baseline patient characteristics.

Variable	Overall (*n* = 340)	No AKI (*n* = 295)	AKI (*n* = 45)	*p* Value
**Demographics**				
Age, years	74.26 ± 10.27	75.0 ± 9.14	69.42 ± 15.06	0.019
Male gender	69.1% (235)	69.5% (205)	66.7% (30)	0.730
BMI (kg/m^2^)	27.91 ± 5.03	27.76 ± 4.99	28.90 ± 5.26	0.156
Obesity (BMI ≥ 30)	29.4% (100)	28.5% (84)	35.6% (16)	0.380
**Medical history**				
Hypertension	92.4% (314)	92.5% (273)	91.1% (41)	0.762
Hyperlipidemia	63.5% (216)	62.4% (184)	71.1% (32)	0.319
Smoking				0.887
Current	18.8% (64)	19.0% (56)	17.8% (8)	
Prior	29.7% (101)	29.2% (86)	33.3% (15)	
Never	51.5% (175)	51.9% (153)	48.9% (22)	
Diabetes	62.1% (211)	61.4% (181)	66.7% (30)	0.621
Insulin depending	36.2% (123)	35.3% (104)	42.2% (19)	0.187
Coronary artery disease	42.9% (146)	42.0% (124)	48.9% (22)	0.421
Congestive Heart Failure	22.9% (78)	21.0% (62)	35.6% (16)	0.037
Prior MI	16.5% (56)	15.9% (47)	20.0% (9)	0.518
Atrial Fibrillation	34.7% (118)	34.2% (101)	37.8% (17)	0.737
Cerebrovascular disease	3.8% (13)	3.4% (10)	6.7% (3)	0.392
Stroke	10.0% (34)	9.2% (27)	15.6% (7)	0.185
Dementia	3.2% (11)	3.7% (11)	0	0.371
Malignancy	14.1% (48)	12.9% (38)	22.2% (10)	0.107
COPD	14.1% (48)	13.2% (39)	20.0% (9)	0.249
**Medication**				
Aspirin	70.3% (239)	71.9% (212)	60.0% (27)	0.116
Clopidogrel	60.6% (206)	60.0% (177)	64.4% (29)	0.626
Anticoagulants	40.0% (136)	38.6% (114)	48.9% (22)	0.196
Statins	70.6% (240)	72.5% (214)	57.8% (26)	0.053
Other lipid lowering drug	5.0% (17)	4.7% (14)	6.7% (3)	0.480
ACE inhibitor/ARB	75.9% (258)	78.6% (232)	57.8% (26)	0.004
Beta-blocker	72.1% (245)	72.2% (213)	71.1% (32)	0.860
Other antihypertensive drug	68.2% (232)	70.5% (208)	53.3% (24)	0.026
NSAR	16.5% (56)	15.9% (47)	20.0% (9)	0.518
Antibiotics	12.1% (41)	11.2% (33)	17.8% (8)	0.219
Neprotoxic agents *	1.2% (4)	1.0% (3)	2.2% (1)	0.435
Active chemotherapy	0.6% (2)	0.7% (2)	0	1.000
Immunosuppressants	7.4% (25)	6.8% (20)	11.1% (5)	0.352
**Clinical Symptoms**				
Claudicants	45.6% (155)	47.8% (141)	31.1% (14)	0.038
CLI	54.4% (185)	52.2% (154)	68.9% (31)	0.038
Rutherford class 4	15.9% (54)	15.3% (45)	20.0% (9)	0.389
Rutherford class 5	23.8% (81)	22.7% (67)	31.1% (14)	0.259
Rutherford class 6	14.7% (50)	14.2% (42)	17.8% (8)	0.503
Baseline chronic kidney disease				
KDIGO 1	0.9% (3)	1.0% (3)	0	1.000
KDIGO 2	4.7% (16)	5.1% (15)	2.2% (1)	0.705
KDIGO 3	7.1% (24)	7.8% (23)	2.2% (1)	0.225
KDIGO 4	78.8% (268)	80.0% (236)	71.1% (32)	0.175
KDIGO 5	8.2% (28)	5.8% (17)	24.4% (11)	<0.001
Baseline Creatinine level, μmol/L	195.72 ± 102.46	186.08 ± 91.0	258.96 ± 144.35	0.002
Baseline GFR, mL/min/1.73 m^2^	32.07 ± 15.61	33.19 ± 15.75	24.78 ± 12.54	<0.001

Data are reported as % (*n*) or mean ± standard deviation when appropriate. * Aminoglycoside antibiotics, sulfonamides, amphotericin B, or pentamidine. AKI = Acute kidney injury. BMI = Body mass index. MI = Myocardial infarction. COPD = Chronic obstructive pulmonary disease. ACE = Angiotensin-converting enzyme. ARB = Angiotensin receptor blocker. NSAR = Nonsteroidal anti-inflammatory drugs. CLI = Critical limb ischemia. KDIGO = Kidney Disease: Improving Global Outcomes. GFR = Glomerular filtration rate.

**Table 2:** Lesion and procedural characteristics.

Variable	Overall (*n* = 340)	No AKI (*n* = 295)	AKI (*n* = 45)	*p* Value
Prior peripheral vascular intervention *	75.9% (258)	76.6% (226)	71.1% (32)	0.455
Surgical	37.1% (126)	37.3% (110)	35.6% (16)	0.870
Endovascular	68.2% (232)	68.5% (202)	66.7% (30)	0.864
Treated area *				
Aortoiliacal	16.8% (57)	15.3% (45)	26.7% (12)	0.083
Femoropopliteal	76.2% (259)	76.9% (227)	71.1% (32)	0.452
BTK	32.6% (111)	32.5% (96)	33.3% (15)	1.000
Severity				0.183
Stenotic	43.7% (142/325)	45.2% (128/283)	33.3% (14/42)	
Occlusive	56.3% (183/325)	54.8% (155/283)	66.7% (28/42)	
Treatment *				
Diagnostic angiography only	16.2% (55)	16.9% (50)	11.1% (5)	0.391
Covered stent	4.4% (15)	4.4% (13)	4.4% (2)	1.000
POBA	55.6% (189)	53.9% (159)	66.7% (30)	0.147
BMS	22.6% (77)	20.0% (59)	40.0% (18)	0.006
DES	11.2% (38)	10.5% (31)	15.6% (7)	0.313
DCB	48.2% (164)	49.2% (145)	42.2% (19)	0.426
Additional treatments ^†^	27.9% (95)	26.8% (79)	35.6% (16)	0.218
Indication for CO_2_ use *				
CKD stage 3–5	95.9% (326)	95.6% (282)	97.8% (44)	0.703
Prior AKI after ICM application	1.8% (6)	2.0% (6)	0	1.000
Known ICM allergy	6.5% (22)	6.4% (19)	6.7% (3)	1.000
Hyperthyroidism	5.0% (17)	5.1% (15)	4.4% (2)	1.000
Periinterventional hydration (intravenous)	65.6% (223)	69.2% (204)	42.2% (19)	<0.001
Prior CM exposure within 7 days	0.6% (2)	0.7% (2)		1.000
Bailout ICM use	80.6% (274)	81.0% (239)	77.8% (35)	0.685
ICM amount, mL	21.23 ± 14.09	20.92 ± 13.86	23.31 ± 15.60	0.344
Reason for the bailout ICM use ^‡^				
Insufficient image quality	88.0% (234/266)			
CO_2_ intolerance	12.0% (32/266)			
Fluoroscopy time, mm:ss	16:50 ± 13:33	16:17 ± 13:10	20:24 ± 15:37	0.058
Area-dose product Gycm^2^	83.91 ± 78.87	75.38 ± 64.75	139.86 ± 127.35	<0.001
Complication associated with CO_2_	1.8% (6)			
Severe abdominal/leg pain	0.9% (3/340)			
Nausea	0.6% (2/340)			
Vomiting	0.3% (1/340)			
Hypotension	-			
Gas embolism	-			
Procedural Success	92.3 (262/284)	92.7% (227/245)	89.7% (35/39)	0.520

Data are reported as % (*n*), % (*n*/*N* if *N* ≠ 340) or mean ± standard deviation when appropriate. * multiple answers possible. ^†^ Debulking, Lysis, Cutting, Scoring. AKI = Acute kidney injury. BTK = Below the knee. POBA = Plain old balloon angioplasty. BMS = Bare metal stent. DES = Drug-eluting stent. DCB = Drug-coated balloon. CO_2_ = Carbon dioxide. CKD = Chronic kidney disease. AKI = Acute kidney injury. ICM = Iodinated contrast medium.

**Table 3:** Postinterventional AKI.

Variable	Overall (*n* = 340)	No Bailout ICM (*n* = 66)	Bailout ICM (*n* = 274)	*p* Value
Postinterventional AKI (within 7 days)	13.2% (45)	15.2% (10)	12.8 (35)	0.685
No AKI	86.8% (295)	84.5 (56)	87.2 (239)	0.685
AKI Stage 1	9.4% (32)	10.6 (7)	9.1 (25)	0.646
AKI Stage 2	0.9% (3)	1.5 (1)	0.7 (2)	0.478
AKI Stage 3	2.9% (10)	3.0 (2)	2.9 (8)	1.000
Detection of AKI (days)	2.6 ± 1.76			
New onset of dialysis within ≤7 days	2.1% (7)			

Data are reported as % (*n*) or mean ± standard deviation when appropriate. ICM = Iodinated contrast medium. AKI = Acute kidney injury.

**Table 4:** Comparison of adverse events between patients with and without AKI.

Complications	Overall (*n* = 340)	No AKI (*n* = 295)	AKI (*n* = 45)	*p* Value
Periprocedural *				
Pseudoaneurysm	2.1% (7)	2.0% (6)	2.2% (1)	1.000
Bleeding at the puncture site	3.8% (13)	3.4% (10)	6.7% (3)	0.392
TIA	-			
MI	1.2% (4)	0.3% (1)	6.7% (3)	0.008
Acute re-occlusion of target lesion within 24 h	0.9% (3)	1.0% (3)	0	1.000
within 30 days				
Major Amputations	1.8% (6)	1.0% (3)	6.7% (3)	0.033
Death within 30 days	3.5% (12)	3.4% (10)	4.4% (2)	0.664

* multiple events possible. Data are reported as % (*n*). TIA = Transient ischemic attack. MI = Myocardial infarction.

## Data Availability

The data that support the findings of this study are available on request from the corresponding author, TW. The data are not publicly available due to privacy restrictions.

## Supplementary Materials

The following supporting information can be downloaded at: https://www.mdpi.com/article/10.3390/life15071046/s1, Supplemental Figure S1: Study flow chart. Supplemental Figure S2: Confusion matrix illustrating the performance of the logistic regression model in predicting post-contrast AKI. Supplemental Figure S3: ROC curve for model performance evaluation.
