# Supplementary material for: Acute Kidney Injury After Peripheral Interventions Using Carbon Dioxide Angiography—Risk Factors Beyond Iodinated Contrast Media
Source: Life (Basel). 2025 Jun 30;15(7):1046. doi: 10.3390/life15071046 (PMC12299457; doi:10.3390/life15071046)
Supplement: Supplementary file 1 [file life-15-01046-s001.zip › life-3723996-supplementary.pdf]

Supplemental material

Supplemental Figure S1: Study flow chart.

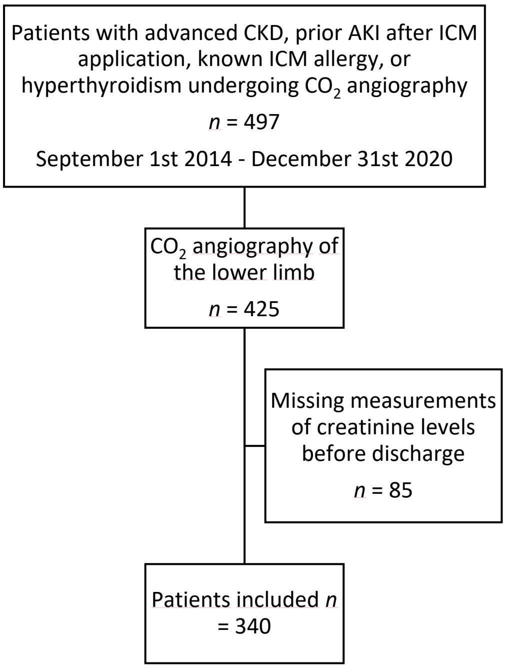

CKD: Chronic kidney disease, AKI: Acute kidney injury, ICM: Iodinated contrast medium, CO_2: Carbon dioxide.

Supplemental Figure S2: Confusion matrix illustrating the performance of the logistic regression model in predicting post-contrast AKI.

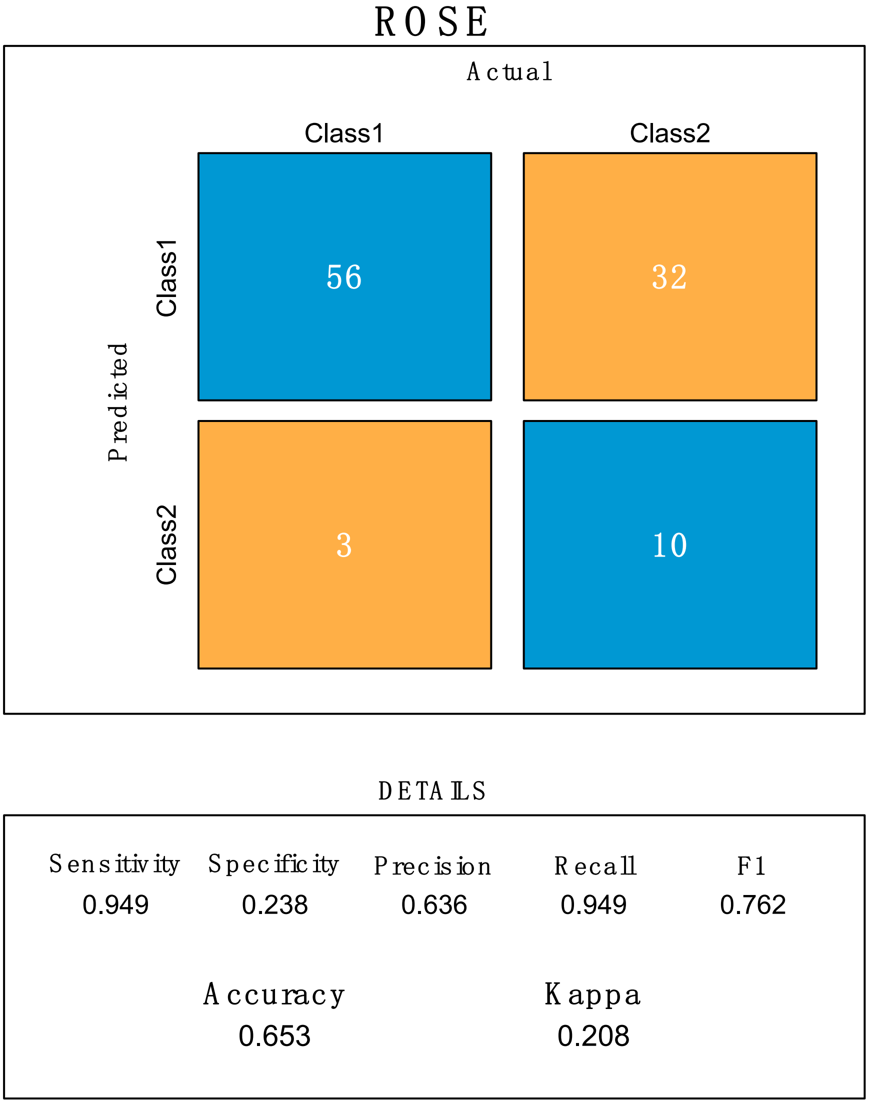

The matrix displays the number of true positives (top left square), false negatives (bottom left square), false positives (top right square), and true negatives (bottom right square). Class 1 represents patients without AKI, while Class 2 represents patients with AKI. Key performance metrics are summarized at the bottom. AKI = Acute kidney injury.

Supplemental Figure S3: ROC curve for model performance evaluation.

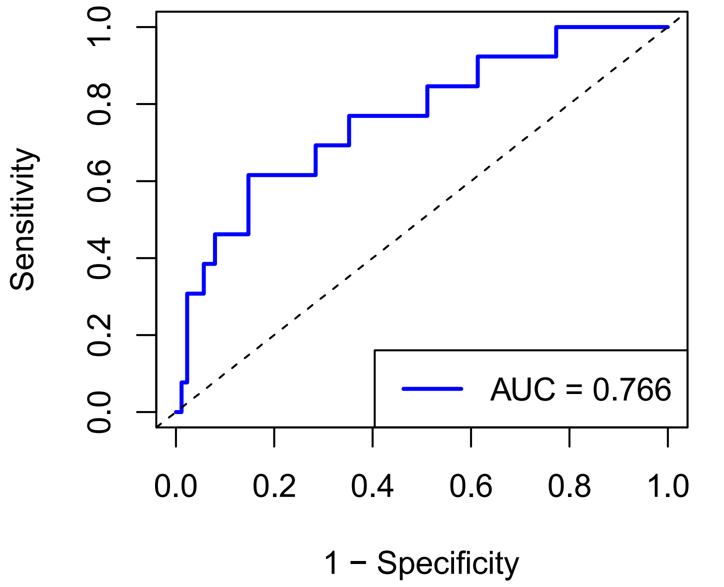

The curve shows the true positive rate (sensitivity) against the false positive rate (1 - specificity) at various thresholds of the model. The area under the curve (AUC) represents the overall performance of the model (0.766). ROC = Receiver operating characteristic.
